# Supplementary material for: Mechanistic insights into the Japanese encephalitis virus RNA dependent RNA polymerase protein inhibition by bioflavonoids from Azadirachta indica
Source: Sci Rep. 2021 Sep 13;11:18125. doi: 10.1038/s41598-021-96917-0 (PMC8437980; doi:10.1038/s41598-021-96917-0)
Supplement: Supplementary file 1 — Supplementary Information. [file 41598_2021_96917_MOESM1_ESM.docx]

**Mechanistic insights into the Japanese Encephalitis Virus RNA dependent RNA polymerase protein inhibition by bioflavonoids from** ***Azadirachta indica***

Vivek Dhar Dwivedi ^1^, Ankita Singh ^2^, Sherif Aly El-Kafraway ^3,4^ , Thamir A. Alandijany ^3,4^, Arwa A. Faizo ^3,4^, Leena Hussein Bajrai^3,5^, Mohammad Amjad Kamal ^3,6,7^, Esam Ibraheem Azhar ^3,4,^*

^1^ Centre for Bioinformatics, Computational and Systems Biology, Pathfinder Research and Training Foundation, Greater Noida, India-201308

^2^ School of Environmental Sciences, Jawaharlal Nehru University, New Delhi, India-110067

**^3^** Special Infectious Agents Unit, King Fahd Medical Research Center, King Abdulaziz University, Jeddah, Saudi Arabia

^4^ Department of Medical Laboratory Technology, Faculty of Applied Medical Sciences, King Abdulaziz University, Jeddah, Saudi Arabia

^5^ Biochemistry Department, Faculty of Sciences, King Abdulaziz University, Jeddah, Saudi Arabia

^6^ Enzymoics, 7 Peterlee Place, Hebersham, NSW 2770; Novel Global Community Educational Foundation, Australia

^7^ West China School of Nursing / Institutes for Systems Genetics, Frontiers Science Center for Disease-related Molecular Network, West China Hospital, Sichuan University, Chengdu 610041, Sichuan, China.

***** Correspondence: eazhar@kau.edu.sa (E.I.A.)

**Results and Discussion**

**S1.1 Structure based virtual screening**

**Table S1.** List of virtual screened bioflavonoids from Azadirachta indica against Japanese Encephalitis Virus RNA dependent RNA polymerase (RdRp) protein.

| **Compound** | **Model ID** | **Energy** | **nRot** |
| --- | --- | --- | --- |
| Gedunin | 1 | -11.6 | 0 |
| Nimbolide | 1 | -11 | 0 |
| Ohchinin Acetate | 1 | -11 | 10 |
| Kulactone | 1 | -10.8 | 3 |
| Limocinin | 1 | -10.6 | 6 |
| Nimbinin | 1 | -10.6 | 0 |
| Azadirachtin | 1 | -10.4 | 13 |
| Rutin | 1 | -10.2 | 16 |
| Myricetin | 1 | -10.1 | 0 |
| Azadirachtol | 1 | -10 | 9 |
| Vilasinin | 1 | -10 | 4 |
| Desacetylsalannin | 1 | -9.9 | 8 |
| Nimocinol | 1 | -9.8 | 2 |
| Isomeldenin | 1 | -9.8 | 4 |
| Azadiradione | 1 | -9.8 | 3 |
| Kaempferol-3-O-rutinoside | 1 | -9.7 | 15 |
| Salannin | 1 | -9.6 | 9 |
| Desacetylnimbin | 1 | -9.5 | 7 |
| Salannolide | 1 | -9.5 | 10 |
| Khivorin | 1 | -9.5 | 7 |
| Kaempferol-3-O-Beta-D-glucoside | 1 | -9.5 | 10 |
| Salannol | 1 | -9.3 | 9 |
| Hyperoside | 1 | -9.2 | 12 |
| Nimbandiol | 1 | -9.1 | 5 |
| nimbidiol | 1 | -9.1 | 5 |
| Nimbin | 1 | -9.1 | 8 |
| Isonimbinolide | 1 | -9.1 | 9 |
| Nimbione | 1 | -9 | 1 |
| Zafaral | 1 | -8.8 | 6 |
| Beta-Sitosterol | 1 | -8.8 | 7 |
| Margocinin | 1 | -8.8 | 4 |
| Nimosone | 1 | -8.7 | 2 |
| OhchinolideB | 1 | -8.7 | 8 |
| Quercetin | 1 | -8.6 | 6 |
| Nimbinone | 1 | -8.6 | 1 |
| Kaempferol | 1 | -8.4 | 5 |
| Nimbiol | 1 | -8.4 | 1 |
| Sugiol | 1 | -8.2 | 2 |
| Scopoletin | 1 | -6.7 | 2 |
| Behenic | 1 | -5.2 | 21 |

**S1.2. ADME profiling**

**Table S2**: ADMET profiling for the selected bioactive compound from *Azadirachta indica* as inhibitor against jRdRp protein.

| **Properties** | **Gedunin** | **Nimbolide** | **Ohchinin acetate** | **Kulactone** |
| --- | --- | --- | --- | --- |
| iLOGP | 3.22 | 3.51 | 4.42 | 4.54 |
| XLOGP3 | 4.22 | 2.17 | 4.82 | 6.89 |
| WLOGP | 4.24 | 3.74 | 5.93 | 7.06 |
| MLOGP | 2.56 | 2.28 | 3.35 | 5.63 |
| Silicos-IT Log P | 4.44 | 3.83 | 5.79 | 6.72 |
| Consensus Log P | 3.74 | 3.11 | 4.86 | 6.17 |
| ESOL Log S | -5.4 | -3.94 | -6.39 | -6.79 |
| ESOL Solubility (mg/ml) | 1.93E-03 | 5.30E-02 | 2.64E-04 | 7.35E-05 |
| ESOL Solubility (mol/l) | 4.00E-06 | 1.14E-04 | 4.10E-07 | 1.62E-07 |
| ESOL Class | Moderately soluble | Soluble | Poorly soluble | Poorly soluble |
| Ali Log S | -5.93 | -3.74 | -6.87 | -7.61 |
| Ali Solubility (mg/ml) | 5.64E-04 | 8.57E-02 | 8.63E-05 | 1.11E-05 |
| Ali Solubility (mol/l) | 1.17E-06 | 1.84E-04 | 1.34E-07 | 2.44E-08 |
| Ali Class | Moderately soluble | Soluble | Poorly soluble | Poorly soluble |
| Silicos-IT LogSw | -5.75 | -5.27 | -7.65 | -6.7 |
| Silicos-IT Solubility (mg/ml) | 8.50E-04 | 2.49E-03 | 1.44E-05 | 9.03E-05 |
| Silicos-IT Solubility (mol/l) | 1.76E-06 | 5.35E-06 | 2.23E-08 | 2.00E-07 |
| Silicos-IT class | Moderately soluble | Moderately soluble | Poorly soluble | Poorly soluble |
| GI absorption | High | High | Low | Low |
| BBB permeant | No | No | No | No |
| Pgp substrate | Yes | Yes | Yes | No |
| CYP1A2 inhibitor | No | No | No | No |
| CYP2C19 inhibitor | No | No | No | No |
| CYP2C9 inhibitor | No | No | Yes | Yes |
| CYP2D6 inhibitor | No | No | No | No |
| CYP3A4 inhibitor | No | No | No | No |
| log Kp (cm/s) | -6.25 | -7.61 | -6.81 | -4.17 |
| Lipinski #violations | 0 | 0 | 1 | 1 |
| Ghose #violations | 1 | 0 | 4 | 3 |
| Veber #violations | 0 | 0 | 0 | 0 |
| Egan #violations | 0 | 0 | 1 | 1 |
| Muegge #violations | 0 | 0 | 1 | 1 |
| Bioavailability Score | 0.55 | 0.55 | 0.55 | 0.55 |
| PAINS #alerts | 0 | 0 | 0 | 0 |
| Brenk #alerts | 2 | 2 | 3 | 1 |
| Leadlikeness #violations | 2 | 1 | 3 | 2 |
| Synthetic Accessibility | 6.48 | 6.07 | 7.2 | 5.86 |

**S1.3. Re-docking and intermolecular interaction analysis**

**Table S3:** List of selected bioflavonoids as inhibitors of viral RdRp protein and molecular interaction profiling in the respective docked complexes.

| **S. no.** | **Compounds** | **Re-docking Score**  **(kcal/mol)** | **H-bond** | **π-π stacking/**  ***Salt bridge** | **Hydrophobic** | **Polar** | **Negative** | **Positive** | **Glycine** |
| --- | --- | --- | --- | --- | --- | --- | --- | --- | --- |
| 1. | Gedunin | - 10.4 | Ser^604^, Ile^802^ | -- | Leu^411^, Ala^413^, Val^414^, Ala^475^, Ile^476^, Trp^477^, Tyr^610^, Trp^800^, Ile^802^ | Ser^604^, Thr^609^, Asn^613^, Ser^801^ | Asp^541^, Asp^668^ | Arg^460^, Arg^474^ | Gly^412^, Gly^605^ |
| 2. | Nimbolide | -10.9 | Ser^604^, Ser^801^, Ile^802^ | -- | Leu^411^, Ala^413^, Val^607^, Tyr^610^, Cys^714^, Trp^800^, Ile^802^ | Ser^604^, Gln^606^, Thr^609^, Asn^613^, Ser^666^, Ser^801^, His^803^ | Asp^668^,  Asp^669^ | Lys^404^,  Arg^474^,  Lys^471^ | Gly^412^,  Gly^667^ |
| 3. | Ohchinin acetate | -11.0 | Trp^477^, Ser^604^, Ser^801^, Ile^802^ | -- | Ala^410^, Leu^411^, Ala^413^, Val^414^, Ala^475^, Ile^476^, Trp^477^, Val^607^, Tyr^610^, Cys^714^, Trp^800^, Ile^802^ | Asn^495^, Ser^604^, Thr^609^, Asn^613^, Ser^801^, His^803^ | Asp^668^,  Asp^669^ | Lys^404^, Arg^460^, Arg^474^,  Lys^471^ | Gly^412^, Gly^603^, Gly^605^, Gly^667^ |
| 4. | Kulactone | -10.4 | Asn^613^ | -- | Val^414^, Phe^415^, Ala^475^, Ile^476^, Phe^478^, Tyr610, Trp^800^ | Thr^346^, Ser^604^, Thr^609^, Asn^613^, Ser^666^ | Asp^541^, Asp^668^ | Arg^460^, Arg^474^ | Gly^412^,  Gly^605^, Gly^667^ |
| 5. | Guanosine-5'-triphosphate | -9.0 | Asp^541^ , Asp^668^, Asp^669^, Ser^715^ | Arg^474^, Arg^474^, Lys^463^ | Trp^54^, Tyr^610^, Cys^714^, and Ile^802^ | Ser^604^, Asn^613^, Ser^666^, Ser^715^, Ser^799^, Ser^801^, His^803^ | Asp^541^,Asp^668^, Asp^669^ | Lys^463^, Lys^471^, Arg^474^, Arg^734^ | Gly^667^ |
| 6. | Adenosine triphosphate | -8.6 | Leu^411^, Ala^413^, Val^607^(2), Val^608^, Tyr^610^, Cys^714^ and Ile^802^ | -- | Leu^411^, Ala^413^, Val^607^(2), Val^608^, Tyr^610^, Cys^714^ and Ile^802^ | Ser^604^, Gln^606^, Thr^609^, Asn^613^, Ser^715^, Ser^801^, His^803^ | Asp^668^, and Asp^669^ | Arg^734^, Lys^471^ and Arg^474^ | Gly^412^ and Gly^667^ |


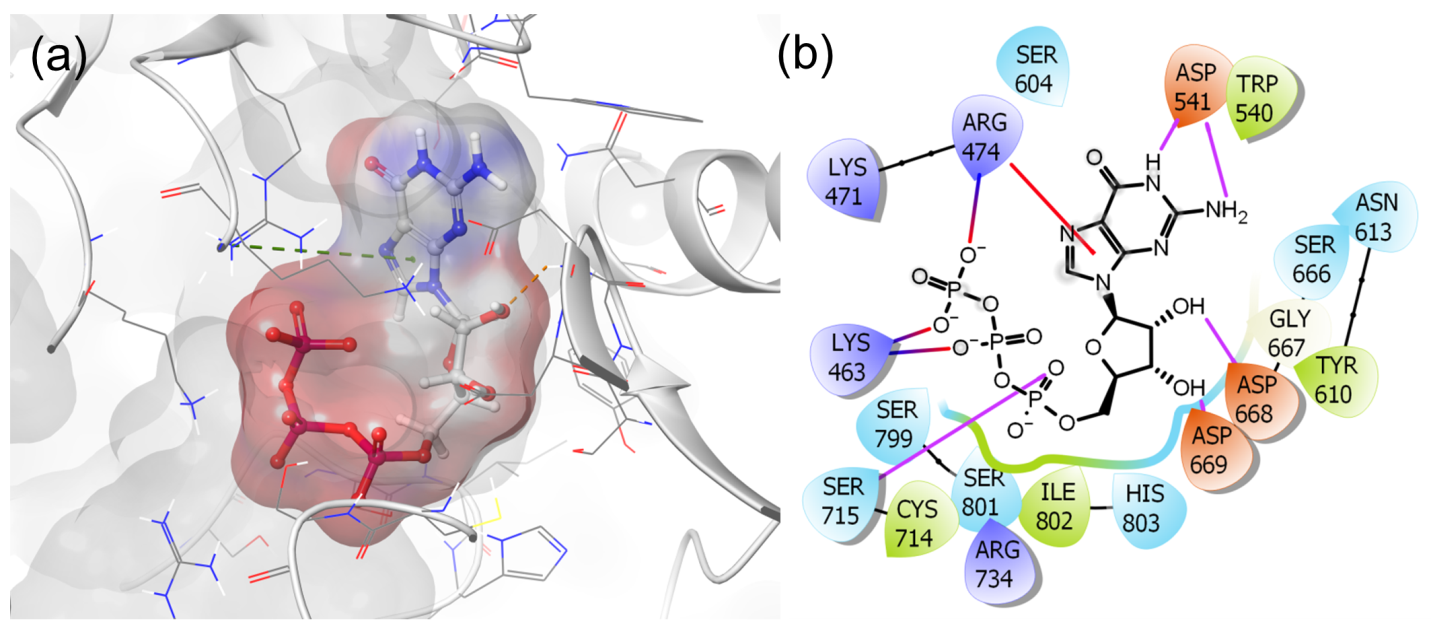


**Figure S1**. 3D and 2D docked poses of the jRdRp-GTP docked complex collected at 4 Å space around the ligand within in the active site of jRdRP protein. In 3D structures, protein surface and ligand surface were rendered based on the alpha-carbon and atomic charge, respectively. While in 2D maps, hydrogen bond formation (pink arrows), Salt bridge (red-violet lines) hydrophobic (green), polar (blue), red (negative), violet (positive), glycine (grey) interactions are logged for docked complexes of jRdRp with selected bioactive compounds.


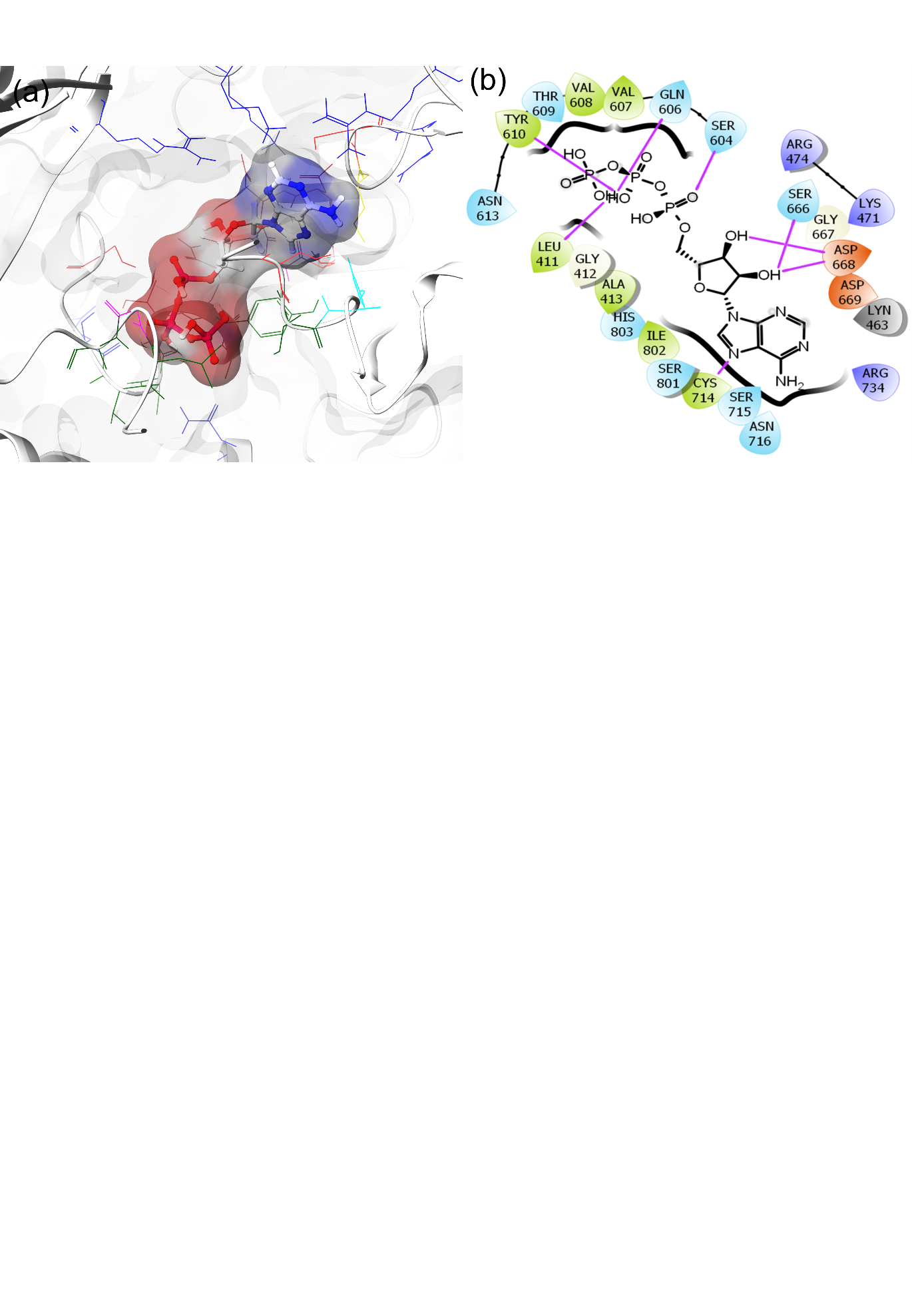


**Figure S2**. 3D and 2D docked poses of the jRdRp-ATP docked complex collected at 4 Å space around the ligand within in the active site of jRdRP protein. In 3D structures, protein and ligand interaction were shown in cartoon mode. While in 2D maps, hydrogen bond formation (pink arrows), Salt bridge (red-violet lines) hydrophobic (green), polar (blue), red (negative), violet (positive), glycine (grey) interactions are logged for docked complexes of jRdRp with ATP.


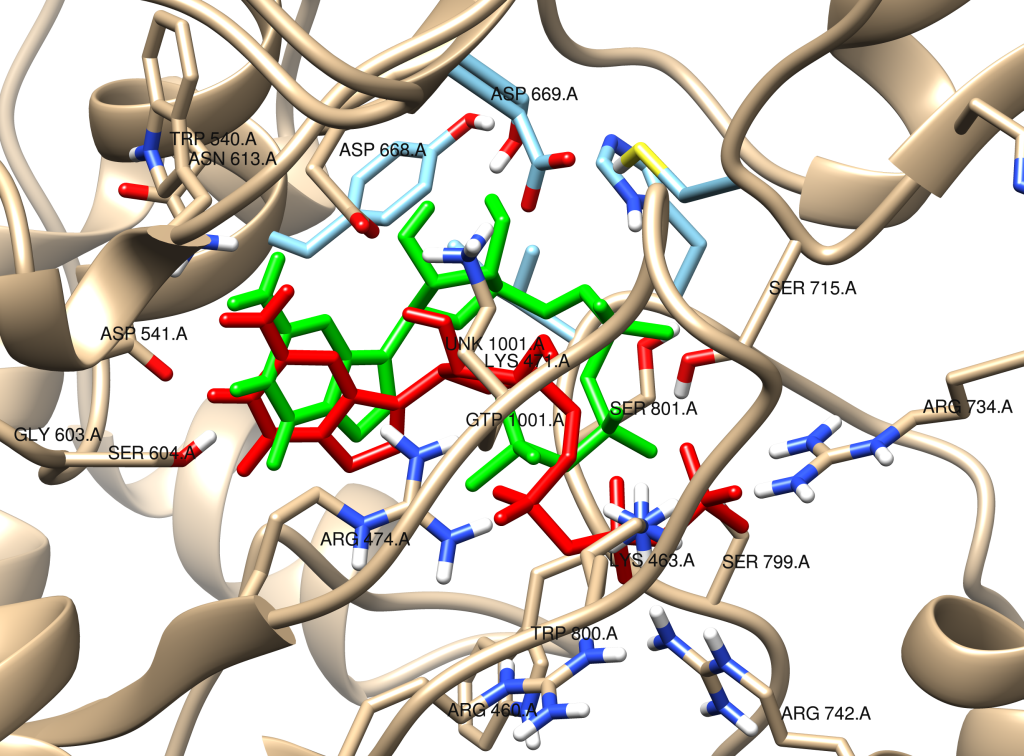


**Figure S3.** Superimposition of jRdRp-GTP re-docked complex with its crystal structure complex

**
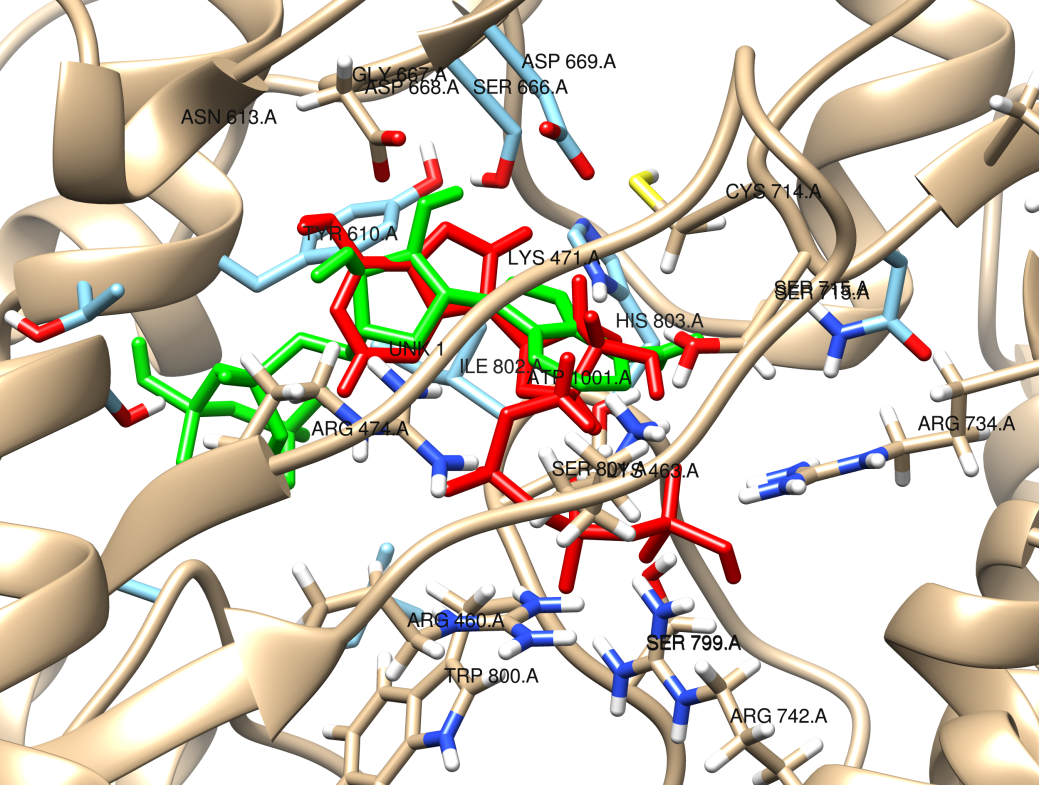
**

**Figure S4.** Superimposition of jRdRp-ATP re-docked complex with its crystal structure complex

**S1.4. Classical molecular dynamics simulation analysis**

**S1.4.1. *RMSD and RMSF analysis***


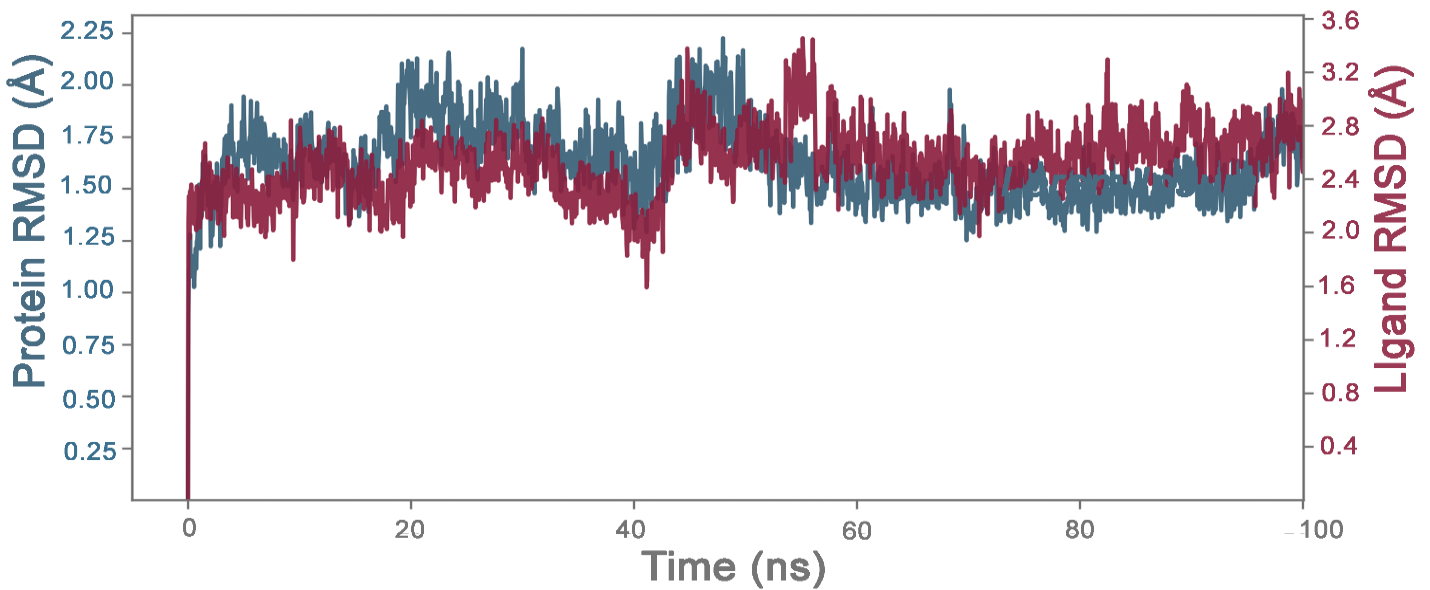

**Figure S5:** RMSD plot for the backbone atoms of RdRp in complex with the reference molecule, i.e. GTP.

**Figure S6:** RMSD plot for the backbone atoms of RdRp in complex with the reference ligand, i.e. ATP.

**
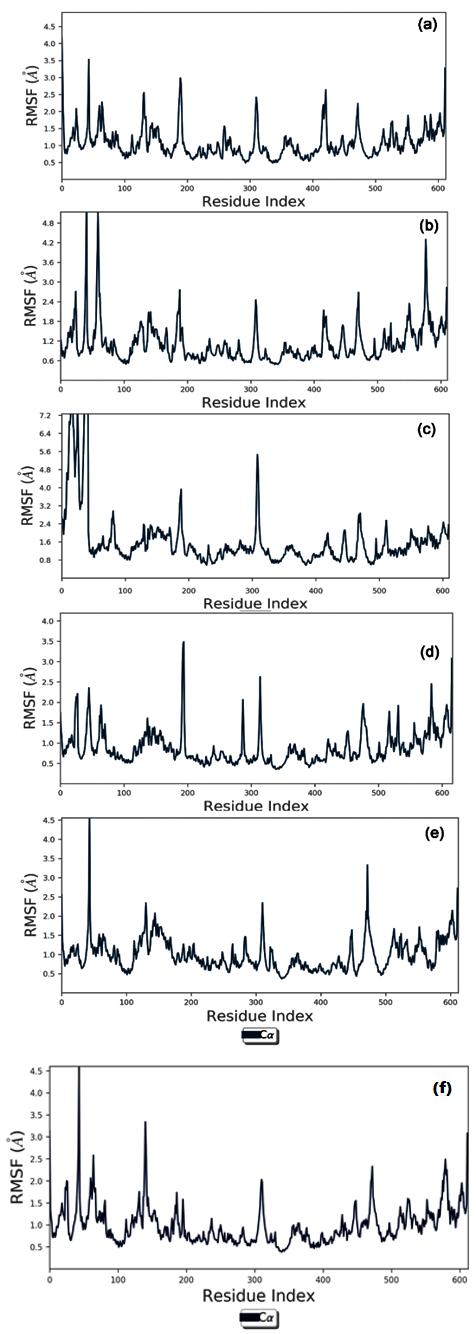
**

**Figure S7.** RMSF plot generated for the jRdRp docked with selected bioactive compounds, i.e., i.e., (a) Gedunin, (b) Nimbolide, (c) Ohchinin acetate, and (d) Kulactone, and reference ligand, viz, (e) GTP, (f) ATP, during 100 ns molecular dynamics simulation interval. Herein, residue number 0-612 are actually residue number from 274-889.


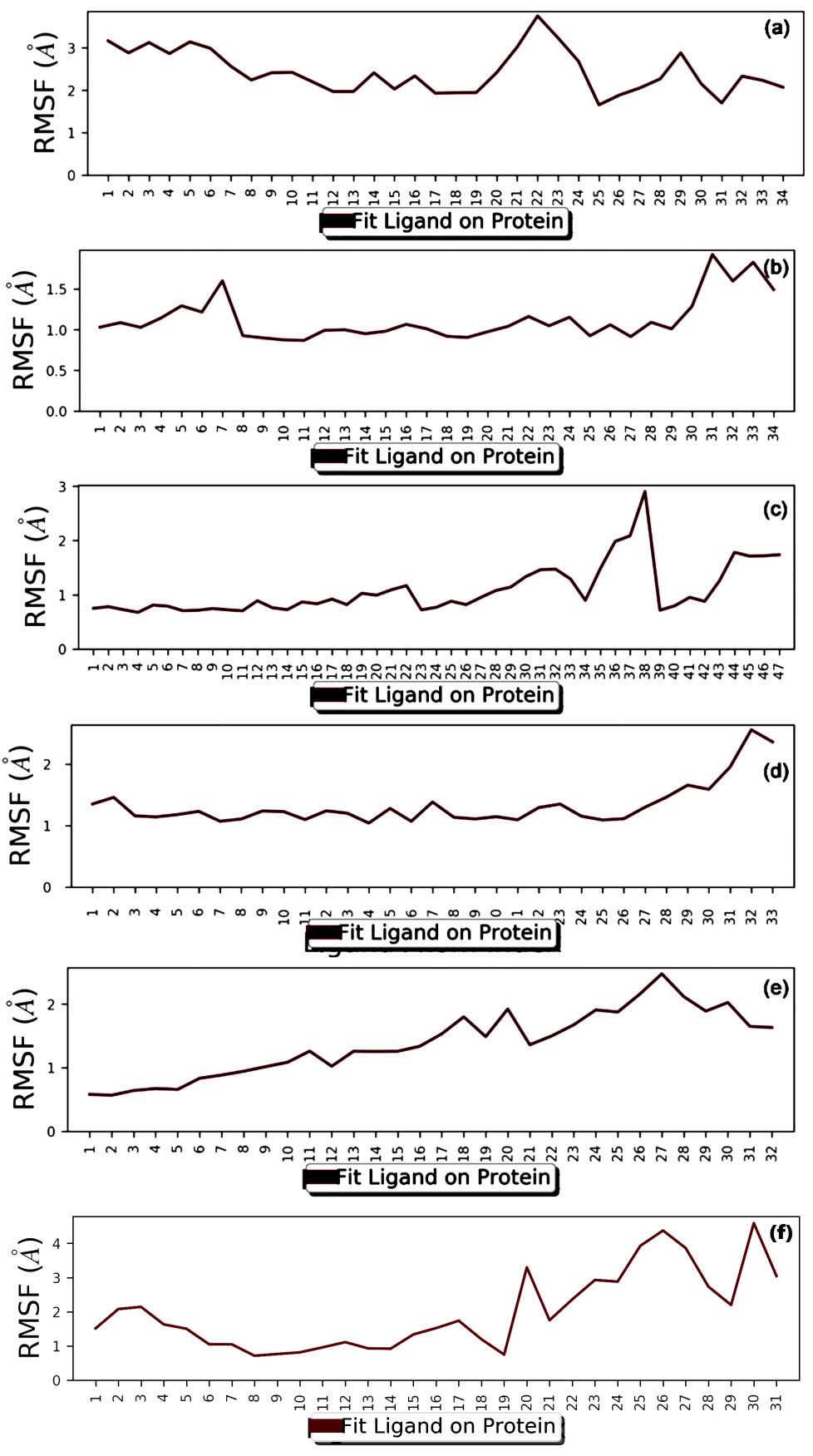


**Figure S8.** RMSF plot generated for the docked bioactive compounds, i.e., (a) Gedunin, (b) Nimbolide, (c) Ohchinin acetate, and (d) Kulactone, and reference ligands, viz, (e) GTP, (f) ATP, fit in the jRdRp protein during 100 ns molecular dynamics simulation interval.

**S1.4.2. *Protein-ligand interaction profiling***





**Figure S9.** Protein-ligand interactions mapping for jRdRp docked with reference compound, i.e. GTP, extracted from 100 ns MD simulations. Herein, values of interaction fractions > 1.0 are feasible as some residues established several interactions of the similar subtype.


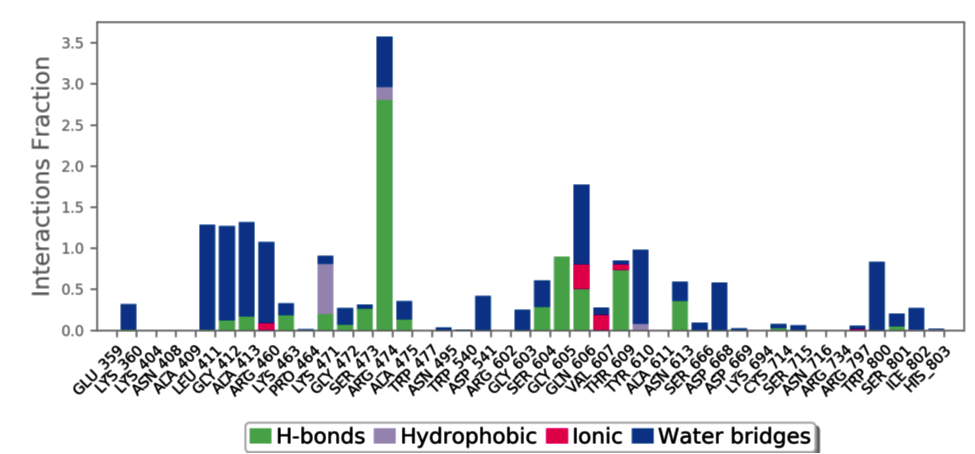


**Figure S10.** Protein-ligand interactions mapping for jRdRp docked with reference compound, i.e. ATP, extracted from 100 ns MD simulations. Herein, values of interaction fractions > 1.0 are feasible as some residues established several interactions of the similar subtype.


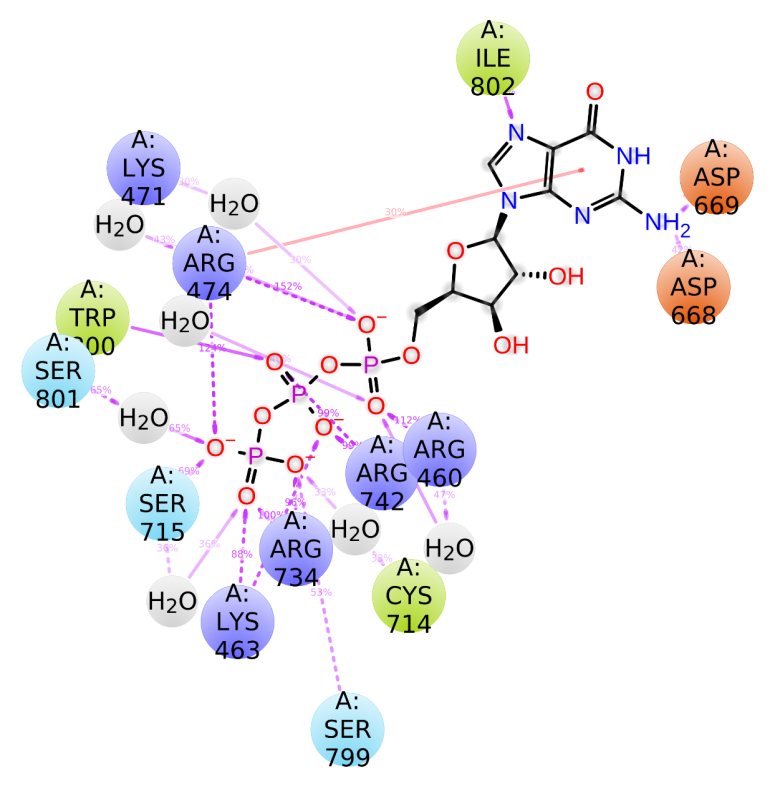


**Figure S11.** Schematic representation for interaction profile of jRdRp-GTP complex extracted at 30% of total 100 ns simulation interval.


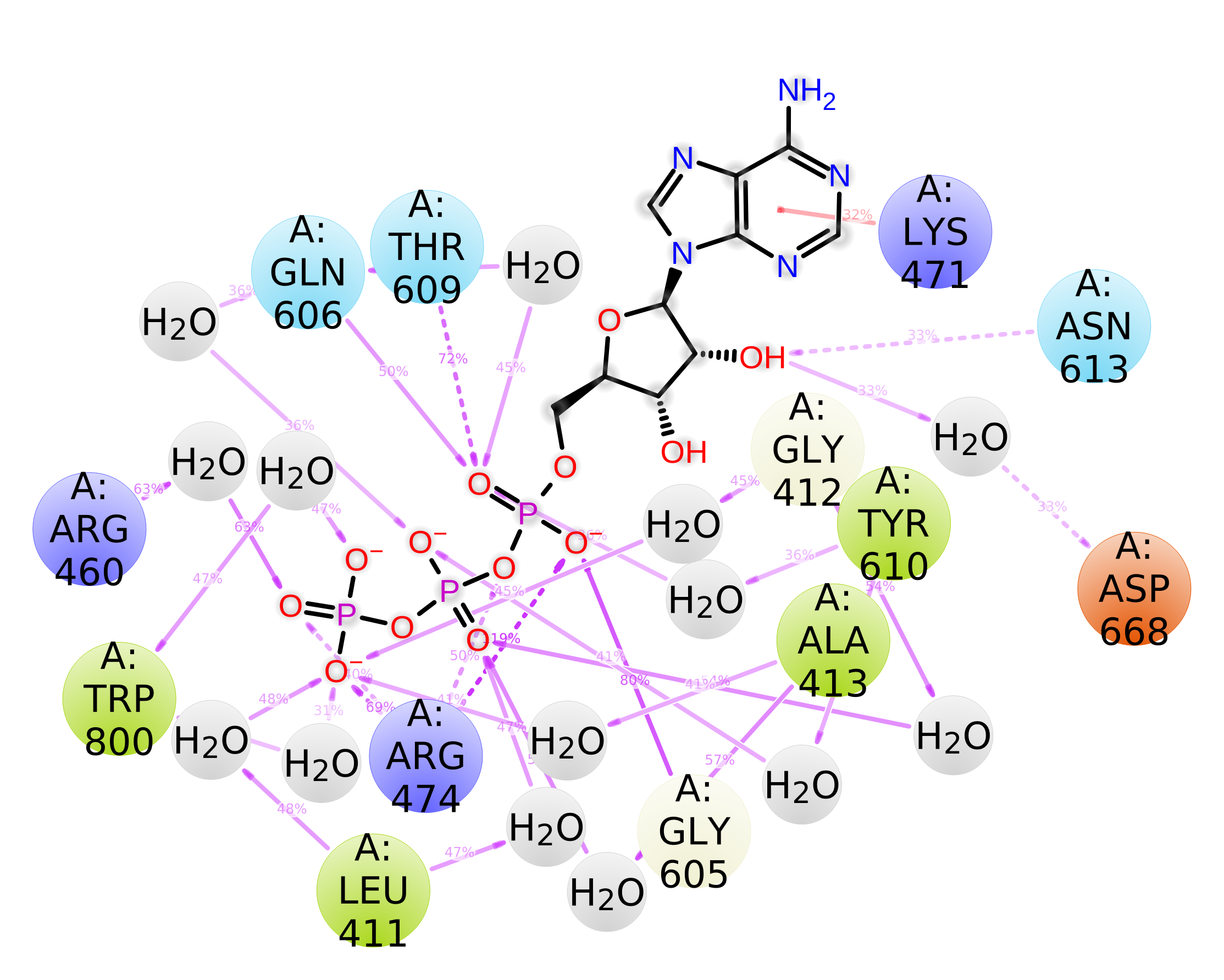


**Figure S12.** Schematic representation for interaction profile of jRdRp-ATP complex extracted at 30% of total 100 ns simulation interval.

**S1.5. Binding free energy analysis**

**Table S4** : Binding free energy and individual dissociation energy components calculated for selected docked complexes of jRdRp protein with bioactive compounds, i.e. (a) Gedunin, (b) Nembolide, (c) Ohchinin acetate, and (d) Kulactone.

| **MM/GBSA**  **Components** | **Energy (kcal/mol)** | | | | | |
| --- | --- | --- | --- | --- | --- | --- |
|  | **jRdRp-Gedunin** | **jRdRp-Nembolide** | **jRdRp-Ohchinin acetate** | **jRdRp-Kulactone** | **jRdRp-GTP** | **jRdRp-ATP** |
| ΔG Bind | -54.54±5.09 | -61.13±3.26 | -61.09±7.08 | -58.65±7.41 | -78.9±9.05 | -33.34±3.61 |
| ΔG_Bind Coulomb_ | -7.27±3.05 | -15.73±5.14 | -8.26±6.35 | -3.96±4.32 | -217.04±21.49 | -117.69±10.48 |
| ΔG_Bind Covalent_ | 3.03±1.23 | 1.15±0.57 | -2.1±1.13 | 1.24±0.68 | 6.79±2.61 | 2.92±0.81 |
| ΔG_Bind Hbond_ | -0.95±0.35 | -0.96±0.54 | -0.54±0.61 | -0.37±0.41 | -17.83±2.05 | -9.42±0.75 |
| ΔG_Bind Lipo_ | -20.03±1.91 | -22.57±2.27 | -16.98±3.91 | -23.5±2.85 | -6.62±0.76 | -2.39±0.27 |
| ΔG_Bind Packing_ | -0.6±0.22 | -1.14±0.32 | -5.03±1.17 | 0±0 | -3.12±0.81 | -1.15±0.27 |
| ΔG_Bind Solv GB_ | 29.36±2.21 | 30.09±2.8 | 31.24±7.04 | 25.45±4.57 | 203.73±14.99 | -127.93±10.83 |
| ΔG_Bind vdW_ | -58.09±2.68 | -51.95±2.27 | -59.42±5.37 | -57.48±4.31 | -44.83±4.41 | -33.66±2.31 |
| Lig Strain Energy | 2.86±0.73 | 1.97±0.8 | 5.56±1.98 | 2.19±1.1 | 6.98±4.03 | -3.81±0.66 |


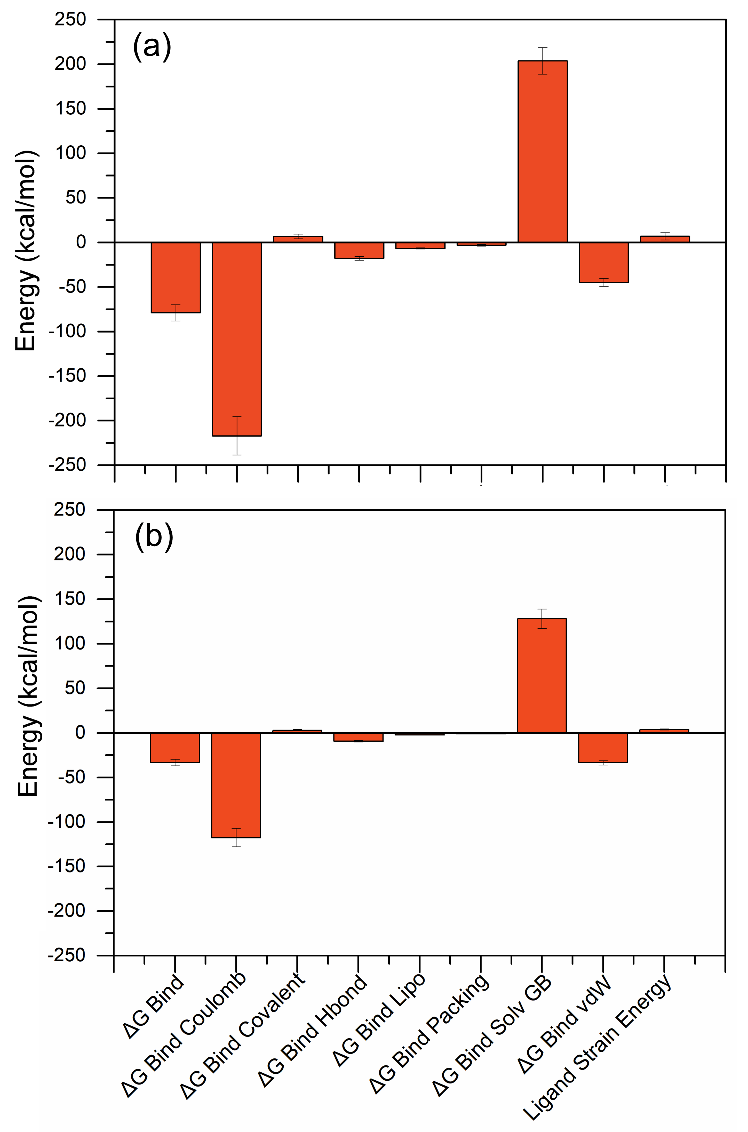


**Figure S13**. Binding free energy and individual dissociation energy components calculation performed for the reference (a) jRdRp-GTP and (b) jRdRp-ATP complexes.
